# Family reintegration of homeless in Maputo and Matola: a descriptive study

**DOI:** 10.1186/s13033-017-0133-7

**Published:** 2017-04-11

**Authors:** Lídia Gouveia, Honório Massanganhe, Flávio Mandlate, Dirceu Mabunda, Wilza Fumo, Ana Olga Mocumbi, Jair de Jesus Mari

**Affiliations:** 1 Department of Mental Health, Ministry of Health-Mozambique, Av. Eduardo Mondlane/Av. Salvador Allende, P.O.Box 1613, Maputo, Mozambique; 2 Faculty of Medicine, University Eduardo Mondlane, Maputo, Mozambique; 3 Department of Psychiatry, Universidade Federal de São Paulo (UNIFESP), São Paulo, Brazil; 4 National Health Institute, Ministry of Health-Mozambique, Maputo, Mozambique

**Keywords:** Homeless, Mental illness, Family reintegration, Mozambique

## Abstract

**Background:**

Homelessness is a global and local social problem with underestimated prevalence. It has been shown to increase the risk of mental illness, raising concerns from mental health providers about the need for effective interventions targeting this population.

**Objectives:**

The aim of this paper is to describe the mental health status of the homeless people in two urban setting in a low-income country, through using standardised clinical and socio-demographic assessments as well assessing potential predictors of family integration versus non-family integration among a group of homeless individuals receiving psychiatric and psychosocial treatment.

**Methods:**

A descriptive study was performed in Maputo and Matola cities between 2008 and 2010. Homeless people with apparent mental illness were mapped and recruited. The participants were referred from community to hospital, using a multidisciplinary treatment model, according to their clinical condition and later entered a family reintegration process.

**Results:**

Seventy-one homeless people were recruited (93.0% male; 80.3% unemployed). The most common diagnosis was schizophrenia and other psychosis (46; 64.8%), followed by mental and behaviour disorder related to substance misuse (21; 29.6%), and intellectual disability (4; 5.6%). Family reintegration was achieved for 53.5% (38 patients). Patients with intellectual disability were less reintegrated and those with disorders related to substance use had better reinsertion in their families (Chi square_*(2)*_ = 6.1; p = 0.047).

**Conclusions:**

Family reintegration was achieved in more than half of participants after hospitalization. Integration was higher in cases of substance misuse, with those with associated intellectual disability being more difficult to reintegrate.

*Trial registration* Trial Registration Number: NCT02936141, date of registration: 14/10/2016, retrospectively registered.

## Background

“Homeless” is a term generally conceptualised to refer to a group of individuals who have no regular access to a decent and conventional housing [1]. However, the concept of decent housing is influenced by local cultural values, hence the difficulty to find a worldwide accepted definition [2]. The homelessness continues to be a global and local social problem, but its true prevalence is underestimated. Urban centres have many people, seemingly homeless, poor and ragged, living all their lives in the street [3]. This is an worldwide and complex phenomenon of big cities that has been increasing in the last decades, particularly due to socio-economic factors [4, 5]. Despite this evolving epidemiology there is scarcity of studies on the prevalence of mental illness in the homeless [4].

Homelessness is known to increase the risk of mental illness, thus raising concerns in mental health providers [6, 7]. In the United States between 33 and 50% of homeless had schizophrenia, and a considerable proportion had substance use disorder, alcoholism and personality disorders [3, 8]. Particularly in children, the high level of exposure of the homeless to abuse and to urban violence can increase risk of developing mental illness [9, 10] in adulthood. On the other hand, schizophrenia can affect cognition and promote impairment of social and professional functioning [11].

Effective intervention strategies may be adapted to promote familial reintegration of homeless people. Intervention models such as the assertive community treatment, were conceived aiming at hospital as well as those community-based approaches [12]. They focus not only on the homeless but also involve the relatives, so as to reduce risk factors for violence, physical abuse and victimization [1, 9].

Between 25 and 50% of homeless populations have some sort of mental disorder in high-income countries. Alcohol dependence, drug addiction and psychotic disorders are among the most common mental health problems identified in previous studies [8]. Little is known about the situation in low middle-income countries, but the impact of the mental disorders among the homeless people is higher in these settings due to shortage of mental health services. Particularly in Africa studies on homeless people are scarce, and thus the size of the problem in the region remains unknown [13].

In Mozambique, although the National Mental Health Program has been providing and implementing services to reduce the gap in treatment of mental disorders, using strategies that include training of psychiatric technicians, there are no specific programs for homeless people. Moreover, there are no specific interventions for the homeless mentally ill in the country, which would combine hospital and community interventions, offered by a multidisciplinary team, composed by psychiatrists, psychologists, psychiatric technicians, nurses and social workers.

A pilot study was designed to evaluate the profile of homeless with apparent mental illness in one urban and one suburban area in a low-income country. Using a referral strategy from community to hospital settings, we aimed to understand the mental health status of the homeless people, as well as to assess potential predictors of family integration, implementing a multidisciplinary approach. Thus, the present work had two main objectives: (a) to characterise the mental health status of the homeless people in Maputo and Matola utilising standardised clinical and sociodemographic assessment; and (b) to look at potential predictors of family integration among homeless submitted to psychiatric and psychosocial treatment.

## Methods

### Target population

The study took place from August 2008 to December 2010, in Maputo city and Matola area, both in the southern region of Mozambique. These cities were chosen because of their accessibility and due to the dimension of the homeless population, representing an urban and suburban area respectively. The homeless were assisted at Infulene Psychiatric Hospital, the main psychiatric health facility in the country admitting patients with mental disorders, as well as providing psychiatric, psychological, occupational and social assistance.

### Study design

Homeless subjects from Maputo city and Matola area were recruited and referred to the Infulene Psychiatric Hospital. The sample comprised homeless people selected by convenience and included in the study using a snowball strategy. Patients with severe physical illness, under the age of 18 who were unable to give informed consent or those who were not in contact with their relatives were excluded. Patients discharged home from Infulene Psychiatric Hospital after being identified and referred by the researchers from homeless communities in Maputo and Matola to hospital, assessed, treated and provided group psychotherapy were eligible for this study.

### Study sample

After psychiatric assessment, patients started the recommended psychotropic treatment. Laboratorial analyses were performed to exclude organic conditions; in positive cases a full clinical examination was requested. Once the symptoms were controlled, the patients were submitted to a psychological evaluation and initiated group psychotherapy sessions and psychopharmacological treatment according to the individual needs. Group psychotherapy sessions included the following: training of social skills (communication, social interaction and assertive behaviors); cognitive stimulation and training of activities of daily living (personal hygiene, hygiene of spaces and standardized mealtimes). All patients were submitted to the same group psychotherapy and occupational therapy. Patients were also exposed to occupational therapy sessions to develop and improve autonomy and self-confidence. In a subsequent phase, group psychotherapy sessions and psychoeducation were also held involving patients’ relatives, as part of family reintegration preparation process. Hospital stay varied from 3 to 6 months. Meanwhile, social workers performed 1–4 home visits to the relatives of the participants so as to create the appropriated environment to the family reintegration process. These visits were done in coordination with the community leaders and the municipal authorities after permission was obtained from patients and their relatives. Throughout these visits, the family social situation, the level of family involvement regarding the therapeutic process, the patient’s health status, and the search information of their family member’s disease were assessed.

We defined family reintegration as the process of return of the homeless from an institution or shelter to its original, extensive or adopted family [14]. In our study, we consider family reintegration as the return of the participant to family of origin after completing the treatment at the hospital level.

We followed every patient for three months after family reintegration. Social workers conducted domiciliar monitoring of the families to provide additional information about disease and its management. Additionally, patients were registered and followed in a health unit near their residence areas as per usual standard of care in Mozambique. The support of clinical staff and social workers was maintained after the study period.

### Data collection method

The diagnosis was established by experienced psychiatrists through a structured clinical interview, the Mini International Neuropsychiatric Interview (MINI), which determines the likelihood of a psychiatric diagnosis by ICD10 [15]. Socio-demographic data was collected using a questionnaire that also included a dichotomized question (yes/no), addressing whether the patient was present or not in the first, second and third monthly visit made by the health professionals. The questionnaire included questions for assessment of social skills adapted from social skills evaluation scale (EEHS) [16], namely: verbal skills, non-verbal skills and conflict resolution that were explored in the three visits.

### Data analysis

Data analysis was performed using the Statistical Package for Social Sciences version 22, for the quantitative data analysis. All the collected information was coded including the identification of patients, relatives, health professionals and community leaders involved in the study. After three months of discharge a health professional was sent to their homes to check if the patient was leaving with their relatives and these was treated as a dichotomy variable. The presence of the patient after three months was defined as “reintegration”.

The present study was waived of approval by the National Bioethics Committee for Health (Ref. 343/CNBS), as it was part of an activity held by the Ministry of Health.

## Results

Eighty-three homeless individuals were identified with apparent mental illness. Of these 12 refused to participate in the study. The 71 homeless recruited (85.5%) had a mean age of 37.83 ± 6.61 years, and 66 (93.0%) were male. Following identification and informed consent all 71 were referred for in-hospital treatment, and after clinical evaluation and social status assessment 38 (53.5%) (n = 38) were reintegrated in their nuclear or foster families. The remaining 33 (46.5%) stayed in the hospital as residents, or returned to the streets.

From the participants in the study 59 (83.1%) were single and 12 (17.0%) married. Fifty-seven (80.3%) homeless were unemployed and 14 (19.6%) had an informal job such car washer, street or market seller, as well as other occasional jobs. The educational level was primary or secondary in 59 (83.1%) and 6 (8.4%) of the homeless had pre-university or university level; the latter have been entirely reintegrated. Regarding their origin 34 (47.9%) street residents were from provinces outside the southern region of Mozambique; Maputo City followed with 30 (42.3%). Before living on the street 32 (45.1%) lived in the suburban neighbourhoods of Maputo City, whilst 24 (33.4%) could not provide reliable data regarding their previous address (Table 1).

**Table 1:** Comparison between socio-demographic data and familial reintegration

Variables	Familial reintegration		
	No n (%)	Yes n (%)	Total N (%)
Gender			
Male	31 (43.7)	35 (49.3)	66 (93.0)
Female	2 (2.8)	3 (4.2)	5 (7.0)
Marital status			
Single	29 (40.8)	30 (42.3)	59 (83.1)
Married/partners	2 (2.8)	4 (5.6)	6 (8.5)
Divorced/separated	2 (2.8)	4 (5.6)	6 (8.5)
Occupational status			
Unemployed	27 (38.0)	30 (42.3)	57 (80.3)
Formally employed	1 (1.4)	4 (5.6)	5 (7.0)
Informally employed	4 (5.6)	5 (7.0)	9 (12.6)
Education			
Illiterate	4 (5.6)	2 (2.8)	6 (8.5)
Primary	21 (29.6)	15(21,1)	36 (50.7)
Secondary	8 (11.3)	15 (21.1)	23 (32.4)
Pre-university	0 (0.0)	5 (7.0)	5 (7.0)
Superior	0 (0.0)	1 (1.4)	1 (1.4)
Place of birth			
Maputo	10 (14.1)	20 (28.2)	30 (42.3)
Maputo province	4 (5.6)	3 (4.2)	7 (9.9)
Outside Maputo	19 (26.8)	15 (21.1)	34 (47.9)
Province residence before living on the street			
Maputo city	9 (12.7)	23 (32.4)	32 (45.1)
Maputo province	4 (5.6)	4 (5.6)	8 (11.3)
Outside Maputo	3 (4.2)	4 (5.6)	7 (9.9)
No information	17 (23.9)	7 (9.9)	24 (33.4)

Schizophrenia and other psychoses were present in 46 (64.8%) participants, mental and behavioural disturbance due to psychoactive substances in 21 (29.6%) and intellectual disability in 4 (5.6%). In general patients did not provide data related to previous treatment; for many the study provided the first contact with mental health professionals. Patients who gave information about their previous illness and hospital admissions reported diagnosis ranging from one to 30 years.

Discharge by drop-out occurred in 28 (39.4%) patients. A statistically significant association ($\chi_{(2)}^{2}$ = *46.1; p* = *0.000)* was verified between type of discharge and family reintegration (Table 2).

**Table 2:** Association between diagnostic, type of discharge and familial reintegration

Variables	Familial reintegration			$\chi_{(df)}^{2}$ *; p value*
	No	Yes	Total	
Diagnosis (ICD-10)				
Schizophrenia and other psychosis	22 (31.0)	24 (33.8)	46 (64.8)	$\chi_{(2)}^{2}$ = *6.1; p* = *0.047*
Intellectual disability	4 (5.6)	0 (0.0)	4 (5.6)	
Alcohol and substance use disorder	7 (9.9)	14 (19.7)	21 (29.6)	
Type of discharge				
Clinical	5 (7.0)	33 (46.5)	38 (53.5)	$\chi_{(2)}^{2}$ = *46.1; p* = *0.000*
Drop-out	26 (36.6)	2 (2.8)	28 (39.4)	
By request	0 (0.0)	3 (4.2)	3 (4.2)	
Transference	2 (2.8)	0 (0.0)	2 (2.8)	

Reintegration in family was associated with the clinical diagnosis ($\chi_{(2)}^{2}$ = *6.1; p* = *0.047*); 24 patients reintegrated (33.8%) had schizophrenia/other psychoses, 14 (19.7%) had mental and behavioural disturbance due to psychoactive substances, and none had intellectual disability. Reintegrated patients’ relatives possessed reasonable to good information about the mental illness in 30 cases and 24 (63.2%) were from low socio-economical level (Table 3). Of these 38 families with reintegrated patients, 23 (60.6%) had reasonable to very good involvement with the patient’s situation.

**Table 3:** Results of the familial reintegration, level of family involvement and family information about the illness

Variable	n (%)
Reintegration	
No	33 (46.5)
Yes	38 (53.5)
Level of family involvement (n = 38)	
Very low	7 (18.4)
Low	8 (21.1)
Reasonable	6 (15.8)
Good	12 (31.6)
Very good	5 (13.2)
Information that the family possesses about the illness (n = 38)	
Weak	8 (21.1)
Reasonable	16 (42.1)
Good	14 (36.8)
Family socio-economical level (n = 38)	
Medium high	2 (5.3)
Medium	10 (26.3)
Low	24 (63.2)
Very low	2 (5.3)

## Discussion

In this study in two areas of southern Mozambique we were able to map, screen, treat and reintegrate homeless with mental illness. The majority of participants were male, single and unemployed. More than half of the sample presented the diagnosis of schizophrenia/other psychoses, followed by mental disorders resulting from addiction, corroborating findings from studies that showed high prevalence of these conditions in people living in the streets [8], and consider them as a driving force for the individual to live in the streets [6].

Like others [17] our results suggests an advantage for patients with schizophrenia/psychosis and mental/behavioural disorders resulting from substance abuse being to being reintegrated, compared to those with intellectual disability. The reduced reintegration of participants with intellectual disability might be explained by the stigma associated to intellectual disability. Intellectual disability being not curable may probably lead to relatives thinking about the patient as a permanent burden. Additionally, feelings of shame due to non-biological explanations for their condition [18] may hamper the possibility of relatives taking their family member back home and assume again the responsibility for their care. Indeed, the conceptual framework for mental disorders may influence relative’s attitudes and determine seeking care pattern. In our setting people attribute intellectual disability to unknown rather than supernatural causes, in contrast with psychosis and epilepsy considered to be have supernatural origins [17].

Socio-demographic factors such as being married, having a nuclear family, being employed, having higher level of education before living in street appear to be protective for homeless people [5] and to improve the chances of reintegration [19, 20]. In our study the reinsertion rate was higher in the female gender, suggesting that women are culturally more protected than men; additionally, having children and living with a partner seem to be protective factors [19] and to influence reintegration in this particular group [20]. The majority of reintegrated patients’ relatives had reasonable to good information about the mental illness; this seems to have contributed to patients’ reintegration and reinforces the importance of family involvement throughout process, including in the pharmacological treatment, rehabilitation and psychotherapy.

Although this is not a causal study the fact that most participants presented mental illness, suggests that this condition may be a driving force (*driver*) for a person to live in the street [6]. Severity of symptoms can lead to neglect of basic personal care (hygiene, seeking for services or resources for social support and health care in the community) [3], besides decreasing the *coping* abilities [5].

Schizophrenia and substance abuse are the two most prevalent disturbances in our sample, like in several epidemiological studies [7, 21], and are risk factor for becoming homeless [21] with strong influence in the socio-family reintegration.

Institutionalization can lead to lack of privacy for residents, but it is known that living in the street may be related to difficulty in socialisation [22]. Our drop-outs were related to the difficulty to adhere to treatment plan, especially in learning social skills. Usually patients were unable to meet schedules and conform to living rules for the group. Additionally, some participants had had problems with the neighborhood before leaving their residences (burning houses or attacking people) leading to their relatives to avoid living with them again. Relatives would rather visit them in the hospital regularly.

Difficult socialisation in hospital environment led to patients abandoning the institution. Dropout from hospital treatment may also be related to the way health providers take care of their clients, some of whom have already created forms of resilience to adapt to life in the street [22] and interact with people around. Moreover, patients discharged following clinical improvement presented higher chances of being reintegrated (46.5%) than those who were discharged at their own request (4.2%) or drop out (2.8%), suggesting that clinical improvement may be an important factor to facilitate family reintegration.

There are several limitations to this study namely lack of preliminary information on the number of homeless and the fact that its findings cannot be generalised to other Mozambican cities. The sample selection procedure chosen (convenience and snowball effect) can induce selection biases related to choosing patients who belong to a specially exposed and stigmatised population. The sample size also constitutes a challenge, considering that the total population of homeless is known. Because we did not have a control group we cannot assess if the success of reintegration was due to the study itself or to unknown confounding factors. Moreover, since only those who were willing to participate were included (or those whose relatives were contacted), homeless with no identified relatives were not represented. Finally, the fact that additional assessment of the global functioning and comorbidities (such as personality and evaluation of traumatic events) had not been included in the study constitutes also a limitation. Despite these limitations our study positively contributed to enhance comprehension regarding potential factors associated to family reintegration that may be considered in strategies to reduce the gap in the treatment of mental illness in Mozambique, specially through utilisation of available resources, and ensuring sustainable implementation of culturally accepted strategies directed to homeless people with mental illness.

## Conclusions

Homelessness coexists with mental illness and is aggravated by low socio-economical level, low school level, unemployment and low healthcare access. Unfavourable socio-demographic factors such as being single or divorced, unemployment, poverty and lack of family involvement influenced family reintegration of the homeless. Schizophrenia, other psychotic disturbances and substance abuse were the conditions with facilitated family reintegration. Reintegration of the homeless that can be achieved with relatively inexpensive integrated approaches, using locally available resources, may help reduce the number of homeless people with mental disturbance in the streets. There is need for research to better understand homelessness and elaborate tailored and culturally adapted psychosocial interventions with multisectoral involvement to reduce the gap in mental care in low-income settings.

## References

[1] Patra S, Anand K (2008). Homelessness: a hidden public health problem. Indian J Public Health.

[2] Dennis DL (1991). A decade of research and services for homeless mentally ill persons. Where do we stand?. Am Psychol.

[3] Shipley SL, Tempelmeyer TC (2012). Reflections on homelessness, mental illness, and crime. J Forensic Psychol Pract.

[4] Scott J (1993). Homelessness and mental illness. Br J Psychiatry.

[5] Crane M (1998). The associations between mental illness and homelessness among older people: an exploratory study. Aging Ment Health.

[6] Sullivan G, Burnam A, Koegel P (2000). Pathways to homelessness among the mentally ill. Soc Psychiatry Psychiatr Epidemiol.

[7] Folsom DP (2005). Prevalence and risk factors for homelessness and utilization of mental health services among 10,340 patients with serious mental illness in a large public mental health system. Am J Psychiatry.

[8] North CS (1997). A diagnostic comparison of homeless and nonhomeless patients in an urban mental health clinic. Soc Psychiatry Psychiatr Epidemiol.

[9] Mello AF (2014). Exposure to maltreatment and urban violence in children working on the streets in Sao Paulo, Brazil: factors associated with street work. Rev Bras Psiquiatr.

[10] Maciel MR (2013). Children working on the streets in Brazil: predictors of mental health problems. Eur Child Adolesc Psychiatry.

[11] Zaytseva Y (2013). Neurocognitive functioning in schizophrenia and during the early phases of psychosis: targeting cognitive remediation interventions. Biomed Res Int.

[12] Marshall M, Lockwood A (2000). Assertive community treatment for people with severe mental disorders. Cochrane Database Syst Rev.

[13] Fekadu A (2014). Burden of mental disorders and unmet needs among street homeless people in Addis Ababa. Ethiopia. BMC Med.

[14] Siqueira AC, Zoltowski AP, Giordani JP, Otero TM, Dell’Aglio DD (2010). Processo de reinserção familiar: estudo de casos de adolescentes que viveram em instituição de abrigo. Estud Psicol.

[15] Sheehan DV (1997). The validity of the Mini International Neuropsychiatric Interview (MINI) according to the SCID-P and its reliability. Eur Psychiatry.

[16] Bandeira M (2002). Escala de Avaliação da Competência Social de Pacientes Psiquiátricos através de Desempenho de Papéis. Aval Psicol.

[17] Patel V, Simbine AP, Soares IC, Weiss HA, Wheeler E (2007). Prevalence of severe mental and neurological disorders in Mozambique: a population-based survey. Lancet.

[18] Tilahun D, Hanlon C, Fekadu A, Tekola B, Baheretibeb Y, Hoekstra RA (2016). Stigma, explanatory models and unmet needs of caregivers of children with developmental disorders in a low-income African country: a cross-sectional facility-based survey. BMC Health Serv Res.

[19] Marjorie J, Winkleby M (1996). Mental health problems of homeless women and differences across subgroups. Amti Rev Public Health.

[20] Rich A, Clark C (2005). Gender differences in response to homelessness services. Eval Program Plan.

[21] Foster A, Gable J, Buckley J (2012). Homelessness in Schizophrenia. Psychiatr Clin N Am.

[22] Padgett DP (2008). Social relationships among Persons who have experienced serious mental illness, substance abuse and homelessness. Am J Orthopsychiatry.

